# HEMOPEXIN’S RELATIONSHIP WITH MUSCLE QUALITY AND COGNITIVE FUNCTION IN OLDER ADULTS WITH COGNITIVE IMPAIRMENT

**DOI:** 10.1093/geroni/igad104.3389

**Published:** 2023-12-21

**Authors:** Derong Zeng, Xiang Qi, Bei Wu, Teruaki Kawasaki, Ichiro Akiguchi, Ayae Kinoshita

**Affiliations:** Kyoto University, Kyoto, Kyoto, Japan; New York University, New York City, New York, United States; New York University, New York City, New York, United States; Kyoto Clinical and Translational Research Center for Neurocognitive Disorders, Kyoto, Kyoto, Japan; Kyoto Clinical and Translational Research Center for Neurocognitive Disorders, Kyoto, Kyoto, Japan; Kyoto University, Kyoto, Kyoto, Japan

## Abstract

The imminent increase in Alzheimer’s disease (AD) and dementia increases the need for effective prevention strategies. Increasig attention has been paid to exercise as a prevention strateg to promote cognitive health. Recently, the link between hemopexin which is secreted by atrophied skeletal muscle and cognitive function maintenance has been studied, but evidence remains inconclusive.This study examined the relationship between hemopexin, muscle mass, muscle quality (Phase Angle, PhA), and cognitive function (measured by Mini-Mental State Examination) among 377 cognitively impaired older adults (mean age = 83.5 years).TANITA (MC-780A-N) was used to measure body composition. The concentration of hemopexin in human plasma samples was measured using the HPX (Human) ELISA Kit (KA0481). In Pearson correlation analysis, hemopexin showed no significant correlation with muscle mass, quality, or cognitive function. Multivariate regression analysis revealed that lower PhA is significantly associated with poorer cognitive function (t-value=2.012, p< 0.05), but hemopexin and muscle mass were not significant. An interaction analysis indicated that the relationship between hemopexin and muscle quality was modified by sex (t-value=2.052, p< 0.05). In the multivariate analysis for females, hemopexin correlated negatively with muscle quality (t-value=-2.128, p< 0.05). This study shows that hemopexin, though not affecting cognitive function or muscle mass directly, may impact muscle quality, especially considering the link between AD in older women and atrophied muscle with hemopexin secretion. Hence, interventions focused on muscle quality might improve cognitive function in older individuals with cognitive impairment, possibly preventing dementia onset. Further studies are warranted to solidify these connections.

